# Decoupling of Medical Public–Private Partnership Efficiency and Pollution to Improve Public Health: A Three-Stage DEA Analysis

**DOI:** 10.3389/fpubh.2021.711084

**Published:** 2021-09-06

**Authors:** Chien-Chi Chu, Zhi-Hang Zhou, Bin Sun, Zhan-Jie Wen, Yu-Yang Ma

**Affiliations:** ^1^Business School, Foshan University, Guangdong, China; ^2^School of Internet Finance and Information Engineering, Guangdong University of Finance, Guangdong, China

**Keywords:** public-private partnership, efficiency, decoupling effect, data envelopment analysis, mortality rate

## Abstract

This study investigates the spatiotemporal evolution of the efficiency of medical public–private partnerships (PPPs) and the decoupling of environmental pollution to promote public health, balanced development, and environmentalism. Based on the 2011–2020 data of medical PPPs in China, the results of a three-stage data envelopment analysis (DEA) and decoupling model show that, firstly, the spatiotemporal evolution of PPP efficiency of China in healthcare has forward periodic twists and turns, and alternating peaks and valleys, which fall into two stages: extensive development, and transformation and upgrading. Secondly, this development is either a type of stable, steady or a surge increase. Thirdly, PPP efficiency and environmental pollution show a weak decoupling state. That is, Northeast China (NEC) and Southwest China (SWC) are in a state of increasing connection, whereas Northwest China (NWC) is in an expanding negative decoupling state. The remaining regions are in a weak decoupling state. This study recommends the mode of ecology-oriented development (EOD) to promote a high-quality, integrated development of PPPs in medicine and healthcare that are especially conducive to a “green economy.” There should be a more coordinated development across regions in China as well.

## Introduction

In China, the “14th 5-Year Plan” was drawn to especially achieve the second centenary goal of China. It establishes the specific ideas, goals, tasks, requirements, and measures for the long-term economic and social development of country. Socioeconomic development planning is inseparable from infrastructure and public services, and this view is reaffirmed in the plan. In this regard, the public–private partnership (PPP) model is identified for its use in an innovative means to enable government and social capital cooperation in infrastructure and public services. It helps accelerate construction and improves both quality and efficiency.

In this study, PPP in medicine refers to the cooperation between government or public medical institutions and social capital to establish hospitals, community medical services, and elderly care, among others, through franchising, service purchase, and equity cooperation. In a narrow sense, medical PPP entails the establishment of non-profit hospitals.

The main advantages of the PPP model are that it helps: achieve higher economic efficiency and value-for-money (VFM); increase investment sources for infrastructure projects; and improve the quality of infrastructure and public services to provide better services for society.

The sudden COVID-19 outbreak in 2020 is expected to continue to have a significant negative impact on the global economy and the health of the population. The pandemic has already exposed the shortage of hospitals and medical and health resources in China but has also brought a new opportunity for reforming the medical system in the region, developing the healthcare industry, and expanding the healthcare capacity of China. Traditional models of economic growth are being challenged, and there is a renewed urgency for sustainable development.

China has a growing medical demand, yet there is an acute imbalance between supply and demand. The medical industry cannot rely on government or private funds alone. Greater social capital is urgently needed to invest in expanding medical and health industries.

In the post-pandemic period, economic recovery—both regional and global—necessitates the “greening” of development. There is an urgent dual requirement of compensating the shortcomings and weaknesses and reducing emissions, and this remains a challenge for medical PPP. Realizing PPP projects is thus critical while pursuing low-carbon development. Two questions need to be addressed: firstly, Have the increasing PPP projects achieved their expected efficiency goals? and Do they promote population health? Secondly, will medical PPP projects contribute to the dual goal of carbon peaking in 2030 and carbon neutrality in 2060? How can we realize low-carbon sustainable development? To evaluate the efficiency of medical PPP across China to find an optimal path more accurately, this study combines a three-stage data envelopment analysis (DEA) with a decoupling theory, and fully considers the differences in economic base, industrial structure, and energy consumption structure in the different regions of China. A more scientific and reasonable measure is created to gauge the efficiency of medical PPP projects.

## Literature Review

### Study on PPP Efficiency Measurements

There are varied definitions of “efficiency” in the literature because of the differences in the coverage of cost, equity, and adaptation to the changes in demand. This diversity makes the study of PPP efficiency difficult (1, 2). The resulted findings are often incomparable. The review of Coelli et al. (3) shows that efficiency most commonly refers to production, allocative, and dynamic efficiencies. Production efficiency is also called technical or cost efficiency, and means maximizing the output under the given input conditions; allocation efficiency is the matching of supply and demand; and dynamic efficiency is the balance between the present and the future and refers to the use of new technologies and operating modes to meet present and future needs. “Fairness” is also considered a dimension of efficiency (4–6), especially in infrastructure and public services (although this view is beyond the scope of this paper).

Even under a relatively clear framework of economic efficiency, there are differences in dimensions that are best reflected in the calculation of key indicators and processing methods. Rhys and Tom (6) define the input–output ratio as a measure of efficiency in cross-sectoral cooperation. The ratio's molecule is service expenditure, and the denominator is a comprehensive output index, which is essentially a proxy variable for many qualitative factors. They later define the input–output ratio as a measure of production efficiency, with the numerator as financial expenditure and the denominator as a proxy variable of the output obtained through a questionnaire survey (7) involving many outcome factors.

Even if the input–output ratio is taken as the measurement index of efficiency, the connotation of efficiency is not limited to a narrow sense of efficiency. Calculating the specific index will still entail different choices based on personal preferences, such as covering input and output, cost and result, or the other choices of compromise. Even the same research question may then yield completely different conclusions.

### Decoupling Effect Research

“Decoupling” implies that multiple physical quantities are unrelated. With the accelerating pace of development, the contradiction between economy and environment is increasingly prominent, and decoupling research has gradually become the focus of analysis in this field. The Organization for Economic Cooperation and Development (OECD) (8) first applied it to agricultural economic research. The World Bank introduced the decoupling theory in the analysis of resources and environment, pointing out that the process of reducing environmental pressure due to economic development not only involves dematerialization but also includes depollution. The OECD shows that “decoupling” is a state where economic growth and environmental pollution are no longer correlated, and it can also be said that this breaks the relationship between environmental burden and economic performance. Once it was conceived, this concept has been universally recognized and widely used in China and abroad.

Decoupling, involving economic development, resources and environment, carbon emissions, transportation, mathematics, finance, and other fields, has been widely studied abroad. In one such study, the data from 1991 to 2000 were used to analyze the relationship between economic development, resources, and environment in Lithuania by means of decoupling, and the existing relationship between resource consumption and economy and the existing relationship between environment and economy were called as primary decoupling and secondary decoupling, respectively (9). The relationship between CO_2_ emissions of transportation vehicles and economic growth was analyzed by researchers using the Finland-related data from 1970 to 2002, and the decoupling relationship between CO_2_ and industrial economy was subsequently measured (10). The decoupling relationship between CO_2_ emissions and economic growth in Brazil from 2004 to 2009 was also analyzed by scholars applying the decoupling theory, and scholars concluded that the absolute decoupling of the two can better promote sustainable economic and social development. (11). The decoupling relationship between economic development and CO_2_ emissions was also analyzed by researchers using the data of European countries from 1990 to 2003 (12). The decoupling analysis method is applicable beyond physics and economics. Maas et al. (13) introduced the decoupling theory in mathematics and analyzed the relationship between Malliavin calculus and decoupling inequality. The relationship between CO_2_ emissions and economic growth in Brazil was analyzed by scholars using the decoupling elasticity index, who concluded that the characteristics of carbon itself and the structure of energy consumption were extremely important factors (14). Some scholars use the equilibrium model to find that it is difficult to achieve the absolute decoupling between economic growth and environmental protection, but tax and subsidy growth rate can be used as the tool to adjust it so as to achieve the relative decoupling (15).

The decoupling effect between economic growth and energy consumption used the energy consumption of per capita gross domestic product (GDP) as an important research indicator (16). The relationship between the decoupling of CO_2_ emissions and industrial growth in North African countries from 1990 to 2016 was analyzed by some scholars. The results show that the effect of energy structure plays an important role in decoupling, and it is necessary to formulate the energy policies that are conducive to the use of more renewable energy to promote the decoupling of North African countries (17). Some research extended the application of a decoupling theory to environmental (such as carbon) and social (such as death) issues, and found that it is more difficult to reduce carbon emissions in the transportation sector than it is to reduce traffic-related deaths, and others studied 15 EU member states from 1995 to 2014 and found that the decoupling effect was not controlled by internal drivers of carbon dioxide emissions (18, 19). The decoupling and influencing factors between economic growth and carbon dioxide emissions in Cameroon were assessed from 1990 to 2015 (20). The analysis concluded that Cameroon showed a weak decoupling during the study period, with the population structure playing a negative role while the economic structure promoted decoupling.

The domestic exploration of decoupling theory is mostly centered around the analysis of the relationship between energy, carbon emissions, and economic growth. However, the research scope has been continuously expanded in recent years, to include areas such as industry, construction, and equipment manufacturing. Most research used the relevant data from 1979 to 2008 to study the status quo, stages, and dynamic change characteristics of low-carbon economic development decoupling in Nanjing and concluded that the decoupling state showed fluctuating changes; in the same year, others analyzed the first batch of low-carbon pilot areas and concluded that absolute decoupling is the top priority for achieving low-carbon economic development (21, 22). The conclusions of studies referenced by Li et al. (23), Gong et al. (24), and Yang and Chang (25) show that most regional economic growth and carbon emissions are weakly decoupled. Other studies used the data from 1995 to 2010 to analyze the relationship between the western regional economy and carbon emissions, and showed a weak decoupling relationship for most studied years (25). From an agricultural economy and a carbon emission perspective and based on the perspective of agricultural economy, some scholars have found that the decoupling relationship (26) between agricultural carbon emissions and agricultural economic growth in the whole country shows a gradual improvement process of “expansion connection state → weak decoupling → strong decoupling”; others showed that economic growth and energy carbon emissions are usually relatively decoupled, and found that this state is mainly due to the decoupling between economy and energy consumption (27). Some scholars have combined the Tapio decoupling index method with the Kaya identity and Log Mean Decomposition Index (LMDI) factor decomposition method to analyze the decoupling relationship between carbon emissions from the energy consumption of China and economic growth from 1996 to 2014 and the driving factors, and the results show that the decoupling effect of carbon emissions from energy consumption and economic growth is the best in China in 1996–2000. After 2000, the overall decoupling state has shown “M”-shaped fluctuation characteristics; the dynamic evolution relationship between carbon emissions and export trade decoupling based on the data from 1990 to 2011 was similarly studied (28, 29). In those studies, the two generally showed a weak decoupling relationship, and reducing carbon emissions can significantly promote decoupling.

Some researchers, using the Tapio decoupling index, focused on the 2000–2015 GDP and carbon dioxide emissions data of 30 provinces and cities in China (30, 31) and found that the decoupling status of different industries is significantly different. Among them, the degree of decoupling in most resource-intensive industries is relatively low. About 43 major carbon emitters along the Belt and Road were selected for the study (32), which found that the coordinated development of economic growth and low-carbon emissions should be emphasized in the process of economic development. A few studies measured the decoupling relationship between the carbon emissions from traffic exhaust and industrial economy in China between 1995 and 2012 (33), and showed that the situation was not ideal and clean energy should be continuously sought to improve the emission situation. Other studies used the Tapio decoupling index and the improved LMDI decomposition method to carry out relevant analyses (34, 35). They found that the carbon emission in the equipment manufacturing industry was mainly in a weak decoupling state with the economy, and that industrial growth and carbon emissions were mainly weakly decoupled in 1996–2000, 2006–2010, and 2011–2015, and were mainly linked by an expansion in 2001–2005. Industrial water use and urbanization (36) could also be analyzed based on the decoupling index model, and the conclusion showed that the two were generally decoupled in 2006–2015, and the degree of decoupling in 2011–2015 was higher than that in 2006–2010. Based on “Belt and Road” countries (representing the countries that China has chosen to invest in the infrastructure) data from 2006, 2009, and 2014, the EKC curve relationship between per capita GDP, per capita energy consumption, and per capita CO_2_ has been studied (37). These countries have transformed from negative decoupling to positive decoupling. Some research results show that the Beijing-Tianjin-Hebei region has achieved strong decoupling in terms of energy conservation and emission reduction (38) from 2007 to 2016, and Hebei Province can promote decoupling through industrial upgrading.

The literature studies on China's PPP project efficiency are still nascent. There are only 20 relevant studies to the best of author's knowledge. There is a limited focus on its overall efficiency or evaluation: most studies focus on the specific aspects of PPP projects, such as investment efficiency (39–42), financing efficiency (43–46), and the cooperative efficiency of government and social capital (47–51). Finally, there is no research on the relationship between the efficiency of medical PPP projects and population health and environmental pollution. There is also a lack of empirical evidence from China on the impact of environmental regulation on healthcare PPP productivity, especially in terms of internal mechanism interpretation and testing. This study addresses the gap in the literature by investigating the spatiotemporal evolution of the efficiency of medical PPP projects in China and decouples environmental pollution to determine what decision strategies can promote public health, balanced and green development, and feasible carbon reduction measures.

### Research Methods

#### Three-Stage DEA Method

Data envelopment analysis can solve the problems in evaluating the relative efficiency of the input and output (52). In this study, this method helps analyze the efficiency of medical PPP projects. In the first stage, the *C*^2^*R* model and the super-efficiency DEA model are used to calculate the efficiency value of PPP projects.

Firstly, the *C*^2^*R* model assumes that the production process is in the stage of constant return to scale, as shown in Equation (1):

(1) $$\begin{array}{l} Max\theta_{j_{0}}=\frac{\sum_{r=1}^{s}u_{r}y_{rj_{0}}}{\sum_{i=1}^{m}v_{i}x_{ij_{0}}}\\ s.t.\left\{ \begin{array}{l} \frac{\sum_{r=1}^{s}u_{r}y_{rj_{0}}}{\sum_{i=1}^{m}v_{i}x_{ij_{0}}}\leq 1,\;j=1,2,\cdots ,n\\ \;u_{r}\geq 0,v_{i}\geq 0, \end{array}\right. \end{array}$$

In Equation (1), *x*_*ij*_ represents the *i*^*th*^ input data of the *j*^*th*^ indicator, *y*_*rj*_ represents the *r*^*th*^output data of the *j*^*th*^index, and *v*_*i*_ and *u*_*r*_ represent the weight of the input and output, respectively.

Suppose $t=\frac{1}{\overset{m}{\underset{i=1}{\sum }}v_{i}x_{ij_{0}}},\mu_{r}=tu_{r},\left(r=1,2,\cdots \;,s\right),\omega_{i}=tv_{i},\left(i=1,2,\cdots \;,m\right)$, and then the Charnes–Cooper transformation in model (1) is performed to obtain model (2):

(2) $$\begin{array}{c} Max\sum_{r=1}^{s}u_{r}y_{rj_{0}}=\theta_{j_{0}}\\ \;s.t\{\begin{array}{c} \sum_{i=1}^{m}\omega_{i}x_{ij_{0}}=1\\ \sum_{r=1}^{s}u_{r}y_{rj}-\sum_{i=1}^{m}\omega_{i}x_{ij}\leq 0,j=1,2,\cdots ,n\\ \mu_{r},\omega_{i}\geq 0,r=1,\cdots ,s,i=1,2,m \end{array}\; \end{array}$$

Equivalent transformation and duality processing in **Equation 2** yield **Equation 3:**

(3) $$\begin{array}{c} Min\theta \\ s.t\{\begin{array}{c} \sum_{j=1}^{n}x_{ij}\lambda_{j}\leq \theta_{j_{0}}x_{ij_{0}},i=1,2,\cdots ,m\\ \sum_{j=1}^{n}y_{rj}\lambda_{j}\geq y_{ij_{0}},r=1,2,\cdots ,s\\ \lambda_{j}\geq 0,j=1,2,\cdots ,n \end{array}\; \end{array}$$

In Equation (3), λ_*j*_ is the linear combination coefficient of the decision-making unit. The reference object of the above model is an “optimal” decision-making unit among all decision-making units. The final relative efficiency is less than or equal to 1. Therefore, the relaxation variable $s_{r}^{+}\left(r=1,2,\cdots \;,s\right)$ and the remaining variable $s_{i}^{-}\left(i=1,2,\cdots \;,m\right)$ are introduced to determine whether the DEA is effective according to the value of the sum. Model (3) then becomes

(4) $$\begin{array}{l} \;Min\theta_{j_{0}}-\varepsilon \left(\sum_{i=1}^{m}s_{i}^{-}+\sum_{r=1}^{s}s_{r}^{+}\right)\\ s.t.\left\{ \begin{array}{l} \sum_{j=1}^{n}x_{ij}\lambda_{j}+s_{i}^{-}=\theta_{j_{0}}x_{ij_{0}},\;i=1,2,\cdots ,m\\ \sum_{j=1}^{n}y_{rj}\lambda_{j}-s_{r}^{+}=y_{rj_{0}},\;r=1,2,\cdots ,s\\ \lambda_{j},\;s_{i}^{-},s_{r}^{+}\geq 0 \end{array}\right. \end{array}$$

where ε is called non-Archimedes infinitesimal. If an optimal solution of the above model satisfies $\theta_{o}^{*}=1$ and $s_{r}^{+}=0,\;s_{i}^{-}=0$, then *DMU*_*j*_0__ is called DEA effective; if an optimal solution of **Equation 4** satisfies $\theta_{o}^{*}<1$, then *DMU*_*j*_0__ is called non-DEA effective.

Secondly, when the efficiency value is calculated, the *C*^2^*R* model often shows that multiple decision-making units are relatively effective; thus, it is impossible to compare and analyze the effective decision-making units. To sort multiple effective decision-making units, Andersen (52) proposed to exclude the tested unit from the reference set so that the efficiency value of the effective decision-making unit could be greater than 1, whereas the efficiency value of the non-DEA effective decision-making unit remained the same as the evaluation result of the *C*^2^*R* model. To distinguish the effective decision-making units, the concept of superefficiency is thus employed. The super-efficiency DEA model is given by **Equation 5**.

(5) $$\begin{array}{l} \;Min\theta -\varepsilon \left(\sum_{i=1}^{m}s_{i}^{-}-\sum_{r=1}^{s}s_{r}^{+}\right)\\ \text{s}.\text{t}.\{\begin{array}{c} \sum_{j=1}^{n}x_{ij}\lambda_{j}+s_{i}^{-}=\theta x_{ij_{0}\;},\;i=1,2,\cdots ,m\\ \sum_{j=1}^{n}y_{rj}\lambda_{j}-s_{r}^{+}=y_{rj_{0}},\;r=1,2,\cdots ,s\\ \lambda_{j},s_{i}^{-},s_{r}^{+}\geq 0,\;j=1,2,\cdots j_{0}-1,j_{0}+1,\cdots ,n\; \end{array} \end{array}$$

The input–output slack variables analyzed in the first stage may be affected by external environmental factors, random errors, and internal management factors. These factors are thus estimated using the stochastic frontier analysis (SFA) method in the second stage of DEA, and their effects are eliminated. **Equation 6** is the SFA regression equation, which is represented as follows:

(6) $$S_{ik}=f_{i}(z_{k};\beta_{i})+\nu_{ik}-\mu_{ik}$$

where *S*_*ik*_ is the slack amount (*i* = 1, 2, …, *n*; *k* = 1, 2, …, *n*)of the *i* input of the *k*th decision-making unit; *f*_*i*_(*z*_*k*_; β_*i*_) is the influence of the *i*th environmental variable element on the *k*th input slack variable *S*_*ik*_, in general form: *f*_*i*_(*z*_*k*_; β_*i*_) = *z*_*k*_β_*i*_, *z*_*k*_ = (*z*_1*k*_, *z*_2*k*_, …, *z*_μ*k*_) is the observable environmental variable of the *k*th decision-making unit. β_*i*_ is the possibly estimated parameter of the environmental variable. The joint term *v*_*ik*_ − μ_*ik*_ is a comprehensive error term, where *v*_*ik*_ is the statistical noise, $v_{ik}\sim N\left(0,\sigma_{vi}^{2}\right)$. *u*_*ik*_ is the *management inefficiency*. The distributions $u_{ik}\sim N^{+}\left(\mu_{i},\sigma_{ui}^{2}\right)$, *v*_*ik*_, and *u*_*ik*_ all obey the normal distribution and are independent and uncorrelated.

In the third stage of the DEA, the data are adjusted. These adjusted input data are used as the new input data. The used output data are the original ones. They are again substituted into the *C*^2^*R* model and super-efficiency DEA model to evaluate efficiency value. Therefore, an efficiency value that is not affected by environmental factors and random errors can be obtained.

#### Decoupling Model

The OECD applies decoupling in agricultural studies to analyze the relationship among agricultural policy, agricultural product trade, and market equilibrium. The World Bank uses it in resources and environmental studies to analyze the decoupling of resource consumption, ecological environment destruction, and economic development.

Currently, OECD and Tapio decoupling models are used for a decoupling analysis. The former model is sensitive to the selection of the time base period, and the calculation results of different time base periods vary greatly. The latter model is a type of elastic analysis unaffected by statistical dimensional changes, and the calculation results have strong stability. The current study analyzes the decoupling relationship between medical PPP projects and population health and environmental pollution using the Tapio decoupling model. In the decoupling model of efficiency and environmental pollution in PPP projects, traditional elastic decoupling is suitable for analyzing the evolutionary process of resource and economic development from linking to decoupling. The extended decoupling model takes the evolutionary process of fishery resources and economy from linking to gradual decoupling, as represented by fishery catch quantity.

Based on the elastic model, the decoupling relationship model between the PPP project efficiency and environmental pollution was constructed as follows:

(7) $$E_{ep}=\frac{(PE_{t+1}-PE_{t})/PE_{t}}{(EP_{t+1}-EP_{t})/EP_{t}}=\frac{\Delta PE_{t}/PE_{t}}{\Delta EP_{t}/EP_{t}}$$

where *E*_*CE*_ represents the elasticity index of efficiency and environmental pollution in the projects; *PE*_*t*_ and *EP*_*t*_ represent the efficiency and environmental pollution of the projects in period *t*, respectively; $\frac{\Delta PE}{PE}$ and $\frac{\Delta EP}{EP}$ represent the changes in the efficiency and environmental pollution of the projects, respectively.

The environmental pollution model was based on the healthy production function developed by Grossman (53), and was improved by Gerking and Starley (54) and others after gradually adding pollution factors. The environmental pollution value is

(8) $$\begin{array}{l} y_{it}=\beta_{0}+\beta_{1}x1_{it}+\beta_{2}x2_{it}+\beta_{3}x3_{it}+\beta_{4}x4_{it}+\beta_{5}x5_{it}\\ \;+\beta_{6}x6_{it}+\varepsilon \end{array}$$

In **Equation 8**, *y*_*it*_ represents the measured environmental pollution value of the *i*th region in China at time *t*, *i* = 1, 2, …, 7, *t* = 2011, 2012, …, 2020. β_0_ represents a constant term. *x*1, *x*2, *x*3, *x*4, *x*5, *and x*6 represent each driving factor (selected from output indicators and environmental indicators), whereas β_1_, β_2_, β_3_, *and β*_4_ represent the coefficient of each driving factor. ε is the error term.

## Data Source and Indicator Description

The data in this article were collected from the China Statistical Yearbook, China Statistical Yearbook on Environment, China Census Data, and China Public Private Partnerships Center. The data from Hong Kong, Macau, and Taiwan are temporarily not included in the scope of the study due to collection issues. The data for 2011 are missing and not included in the scope of the study for the time being.

Land, labor, and capital are usually the most basic factors of production from an economic perspective. Based on the literature and considering the availability of input data, the project area (in m^2^) of the medical PPP was selected as the index of land resource input. In terms of capital investment, the total project investment (in ¥10,000) of the medical PPP was selected as the investment index.

Because the main goal of the medical PPP projects is to promote economic growth and population health, GDP per capita (in yuan) and population mortality rate (in percentage) are selected as output indicators.

Given the variables related to population health, the number of days with air quality above level two, which accounted for the proportion (rrb lrb %), total industrial wastewater discharge (in 10,000 tons), total affected area of crops (103 hm^2^), and the number of environmental emergencies (number of times), was selected as environmental indicators (see Table 1 for specific indicators).

**Table 1:** Efficiency evaluation index of medical PPP projects.

**Index**	**Index content**	**Index description**
Investment index	PA	Land element input
	TPI	Capital factor input
Output indicators	GPC	Measure the wealth possessed by the population or the ability to resist social risks
	PMR	Reflecting the death level of the regional population
Environmental indicators	TP	One of the important indicators to describe the air quality. The lower the indicator, the worse the living environment of the population, and the higher the risk of death.
	TD	An important indicator that affects the quality of the water environment
	TC	Affect the amount of food that the population can obtain and increase the risk of death
	NE	The direct environmental factors that increase the mortality rate of the population are often destructive

### Analysis of Empirical Results

#### Spatiotemporal Patterns of Efficiency

##### Time Dynamic Evolution of Efficiency

The MATLAB calculation of the efficiency of medical PPP projects of China from 2011 to 2020 is shown in Table 2. The efficiency of the projects from 2011 to 2020 is characterized by integrity, stage, and periodicity.

**Table 2:** Efficiency of medical PPP projects from 2011 to 2020.

**Period**	**Year**	**Comprehensive efficiency**	**Technical efficiency**	**Scale efficiency**	**Scale reward**	**Super- efficiency**
China's 12^th^ 5-Year Plan (2011–2015)	2011	-	-	-	-	-
	2012	0.504	0.815	0.618	drs	0.504
	2013	0.519	0.889	0.583	drs	0.519
	2014	0.535	0.926	0.578	drs	0.535
	2015	0.608	0.901	0.675	drs	0.608
	average	0.542	0.883	0.614		0.542
China's 13^th^ 5-Year Plan(2016-2020)	2016	0.602	0.928	0.649	drs	0.602
	2017	0.625	0.935	0.668	drs	0.625
	2018	0.857	1	0.857	drs	0.857
	2019	0.667	1	0.667	drs	0.667
	2020	0.682	0.887	0.767	drs	0.753
	average	0.687	0.950	0.722		0.701

The overall efficiency shows an increasing trend and then a decreasing trend from 2011 to 2020. The peak value of efficiency was 0.857 in 2018, and the trough value was 0.504 in 2012. In a specific historical stage, facing the contradiction between the growing material and cultural needs of people and the backward social production, environmental protection serves, or even gives a way to economic development, with a special era and historical inevitability. However, with increasingly prominent ecological and environmental problems, the continuous improvement of environmental awareness and the extensive implementation of comprehensive environmental protection actions, environmental governance system in China has undergone a transformation from the extensive development of “economic construction as the center” to the sustainable development of “economic development bears environmental responsibility.” Therefore, we can see that the efficiency level of medical PPP projects of China's first increases and then decreases from 2011 to 2020.

From the perspective of stages, the 12th 5-Year Plan was marked by an extensive development, with a continuous growth. From 2012 to 2015, the efficiency value of the projects increased by 0.105% or approximately 21%. The 13th 5-Year Plan saw an intense transformation and upgrading, with wave-like rises moving steadily and farther in the circle. The efficiency value first increased from 0.602 in 2016 to 0.875 in 2018, with an increase of approximately 41%. The efficiency was 0.667 in 2019 and 0.753 in 2020.

The curve of the efficiency value of the PPP project development presents a unity of twists and turns from 2011 to 2020, reflecting a cyclical movement of overall forward periodic twists and turns, and alternating peak and valley values.

##### Evolution of the Spatial Pattern of Efficiency

**Figure 1:**
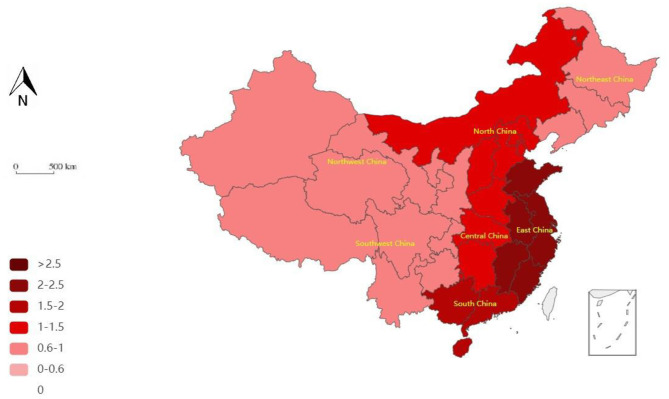
Geographical pattern of the efficiency of medical PPP projects in the seven regions during China's 12th 5-Year Plan period.

**Figure 2:**
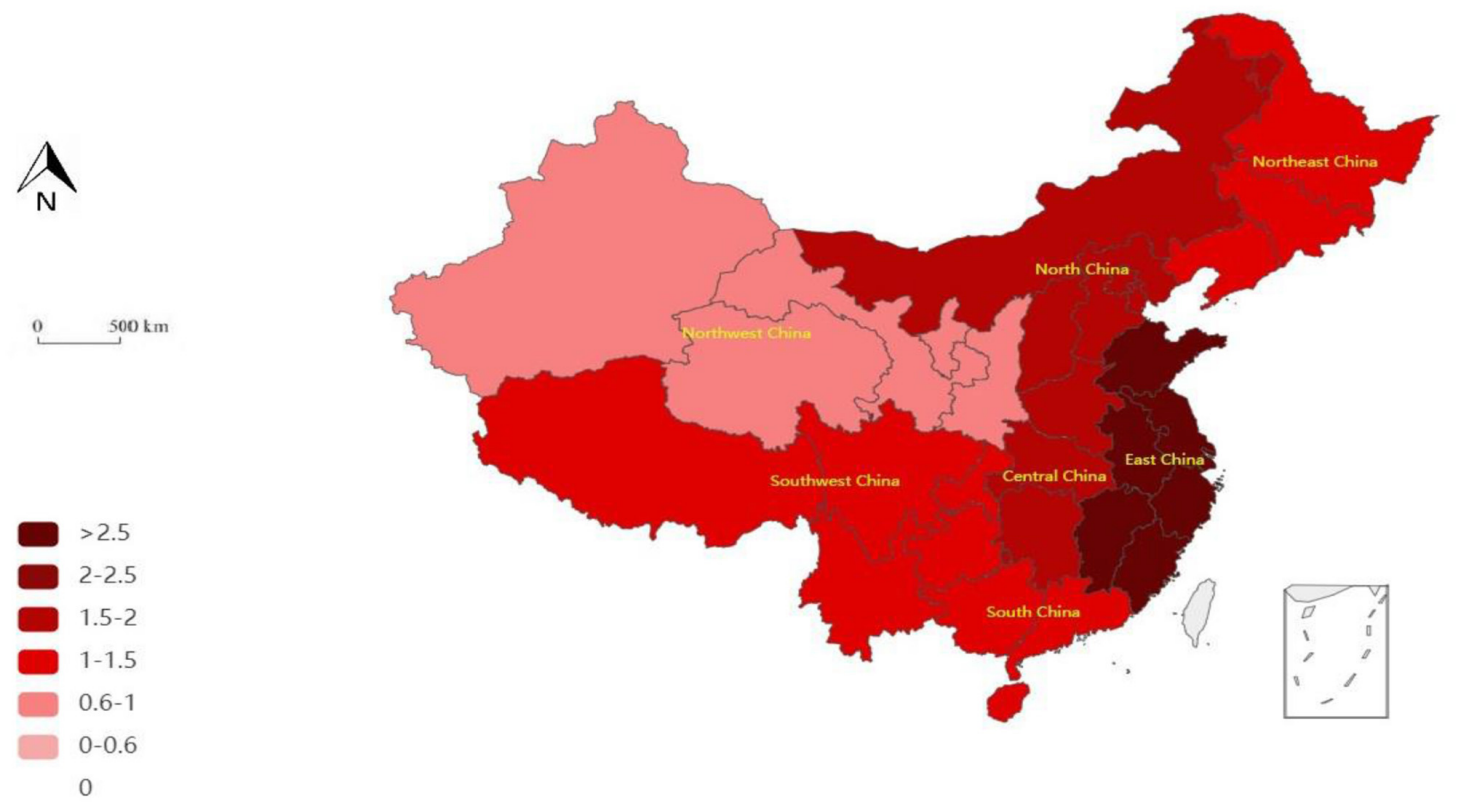
Geographical pattern of the efficiency of medical PPP projects in the seven regions during China's 13th 5-Year Plan period.

Combined with the time evolution of the medical PPP project efficiency value from 2011 to 2020, the spatial pattern of efficiency in the seven regions of China was drawn by selecting cross-sectional data during the 12th and 13th 5-Year Plans. The results are shown in Figures 1, 2. The DEA efficiency values obtained at each stage are divided into different intervals and drawn onto the map, with different efficiency value intervals being represented by different color shades.

Firstly, the overall efficiency shows an increasing trend with a higher trend in the 13th plan than in the 12th plan. China's population structure presents the characteristics of accelerated aging, a decline in labor force population, and an expected arrival of the third baby boom generation. The aging trend increases the demand for medical services, whereas the appearance of the labor force inflection point also increases the need for investment in human capital. In this context, the Chinese government has drawn increased attention to population health and medical resources. As such, in the 13th 5-Year Plan period, the Healthy China strategy was fully implemented and that driving force, led by policy factors, promoted the development of medical PPP projects and their improved efficiency. Secondly, in the 12th and 13th 5-Year Plans, the efficiency values of East China (EC), South China (SC), North China (NC), and Central China (CC) regions were generally higher than those of the Northeast China (NEC), Southwest China (SWC), and Northwest China (NWC) regions; that is, the efficiency of the projects in economically developed regions is more prominent. This shows that the distribution of medical resources remains unbalanced, which affects the efficiency distribution of medical PPP among the regions.

From the perspective of stages, in the 12th 5-Year Plan, the regional ranking of project efficiency is as follows: EC, SC, NC, CC, NEC, SWC, and NWC, in that order. The efficient regions are EC, SC, NC, and CC, whereas the inefficient regions are NEC, SWC, and NWC.

In the 13th 5-Year Plan, the regional ranking of project efficiency is as follows: EC, CC, NC, SC, NEC, SWC, and NWC regions. The efficient regions are EC, CC, NC, SC, NEC, and SWC China, whereas an inefficient region is NWC. Compared with the 12th 5-Year Plan, the overall and regional efficiencies have improved significantly, especially in CC and EC. However, the efficiency in SC marginally decreased. This phenomenon is mainly due to the implementation of the Healthy China strategy in the 13th 5-Year Plan, which includes more detailed road maps and construction guidelines. The report of the 19th National Congress of the Communist Party of China stressed the need to further implement the Healthy China strategy and elevate health to an unprecedented level, drawing a clear blueprint for a healthy China for the general public.

From the perspective of evolution, the seven regions in China can be divided into three types: stable, surge, and steady increase. SC exhibits a stable evolution of efficiency during the entire study period, with minor fluctuations. CC and EC show a surge in efficiency, which reflects a strong growth: the efficiency value increased by more than 0.5 during the study period. Finally, NC, NEC, SWC, and NWC show a steady growth, with an increase in the efficiency value within 0.5.

This spatiotemporal distribution of efficiency was obtained in the first stage of the DEA. In the second stage, the SFA method was used to calculate and decompose the effect of environmental variables, random errors, and internal management inefficiency, and thereafter adjust the initial values of the three input indicators accordingly to analyze all regions using the same measurement standard. If the calculated coefficient is positive, then an increase in the value of the environmental variable increases the input slack variable or decreases the output, thus increasing the waste. This has an adverse effect on environmental efficiency. A negative coefficient indicates that an increase in the environmental variable reduces the input slack variable or increases the output, benefitting environmental efficiency. The relaxation amounts of the two input indicators calculated in the first stage were taken as the explained variables of the regression function, and the explanatory variables were four environmental indicator variables. The relevant variables were input into Frontier version 4.1, and the influence of the four environmental variables on the two input relaxation variables was analyzed using the maximum likelihood method. The results are presented in Table 3.

**Table 3:** Regression adjustment results of second stage.

**Variable**	**PA**	**TPI**
Constant term	4.45^***^ (2.70)	9.03^***^ (3.49)
TP	0.002 (12.45)	0.006^**^ (0.79)
TD	0.000 (−1.30)	0.003^***^ (7.70)
TC	−0.001^***^ (−0.92)	0.004 (1.17)
NE	0.311^***^ (7.13)	0.135^***^ (6.66)
Sigma-squared	6.71 (0.7674)	1.1745 (4.7671)
Gamma	0.98^***^ (52.67)	0.97^***^ (163.66)
Log likelihood	−61.57	1.92
LR	280.91	321.43

** and *** *represents the significance level of 5%, and 1%, respectively; the corresponding t-test values are shown in parentheses*.

As it can be seen from Table 3, air quality is positively correlated with the total investment of medical PPP projects, indicating that the worse the air living environment of the population is, the higher the risk of death of that population will be, and the total investment in medical PPP projects will increase to mitigate the harm caused by air pollution to the health of the population. Additionally, the investments for industrial wastewater and medical PPP projects also show a significant positive correlation, indicating that when the water environment quality is poor, the population death risk increases, and the total number of medical PPP projects also increases to slow down the harm caused by water pollution. Finally, the affected area, combined with medical PPP projects, covers an area of a significant negative relationship, which suggests that if a decline in population survival food is obtained, it can increase the risk of death; to control that, the need to cultivate more farmland arises, which could lead to a reduction in land resources for medical PPP projects. It should be noted that emergencies have a positive and significant relationship with the two input variables, meaning that the higher the number of environmental emergencies is, the more the population mortality rate will increase. To control the population mortality, investments in medical PPP projects will increase.

**Figure 3:**
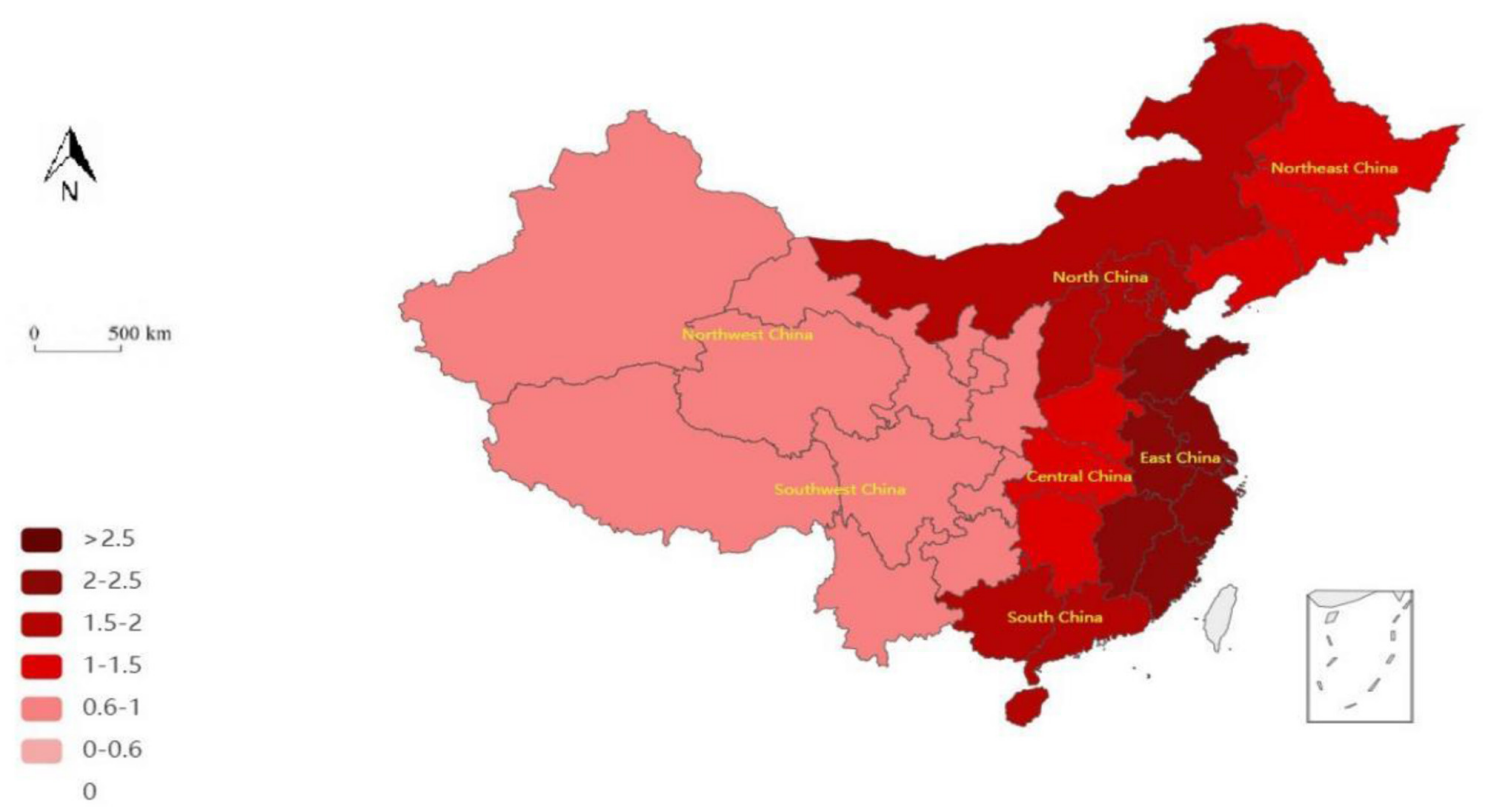
Adjusted geographical pattern of the efficiency of medical PPP projects during China's 12th 5-Year Plan period.

**Figure 4:**
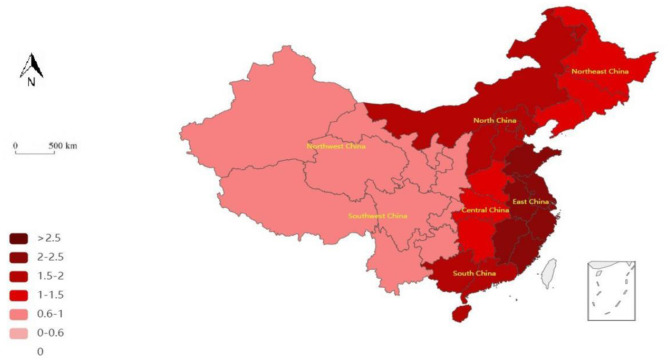
Adjusted geographical pattern of the efficiency of medical PPP projects during China's 13th 5-Year Plan period.

The third stage of the DEA measures the efficiency of the adjusted healthcare PPP. The results are shown in Figures 3, 4.

In general, the period of the 12th and 13th 5-Year Plans shows improved adjusted efficiency. In the former period, there were three inefficient areas before adjustment but only one inefficient area after adjustment. That is, after the adjustment of environmental factors, NEC changed from inefficient to efficient, indicating a positive effect of environmental factors. In the latter period, only NWC was inefficient before the adjustment but became fully efficient after adjusting for environmental factors, confirming our prior result further.

### Analysis of Decoupling Relationship

Infrastructure is known to promote regional economic growth in China but also harms the environment. Such pollution has a restraining effect on further regional economic growth (3, 4, 55, 56). The decoupling of environmental pollution is an idealized process wherein the relationship between the efficiency growth of medical PPP projects and environmental pollution gradually weakens until it disappears. In other words, realizing project efficiency reduces environmental effects. The growth elasticity of the efficiency of environmental pollution is the decoupling of environmental pollution. Thus, elasticity can be the main tool for measuring the environmental protection status of each region. To explore a more accurate decoupling relationship, Tapio decoupling was chosen to divide the decoupling elasticity into eight types: weak, strong, recession type, weak negative, strong negative, expansion type negative, growth connection, and recession connection (see Table 4).

**Table 4:** Decoupling elasticity and states.

**States**		**Environmental pollution**	**Change in project efficiency**	**Decoupling elasticity**
Decoupling	Weak decoupling	>0	>0	0≤D<0.8
	Strong decoupling	<0	>0	D<0
	Recessive decoupling	<0	<0	D>1.2
Coupling	Growth link	>0	>0	0.8≤D≤1.2
	Decline link	<0	<0	0.8≤D≤1.2
Negative decoupling	Weak negative decoupling	<0	<0	0≤D<0.8
	Strong negative decoupling	>0	<0	D<0
	Expansive negative decoupling	>0	>0	D>1.2

Overall project efficiency and environmental pollution show a weak decoupling state from 2011 to 2020 (see Table 5). That is, an increase in efficiency was accompanied by an increase in environmental pollution, but the growth rate of environmental pollution was smaller than that of efficiency. With the increasingly prominent contradiction between environmental problems and economic social development in China, the country's leading political party and government have drawn increased attention to environmental management. Decoupling elasticity in China's 13th 5-Year Plan (2016–2020) was smaller than that in China's 12th 5-Year Plan (2010–2015), highlighting the effectiveness of the former period's transformation and upgrading. For the same increase in efficiency, an increase in environmental pollution in China's 13th 5-Year Plan was less than that in its 12th 5-Year Plan. Since the 12th 5-Year Plan, China's environmental protection has entered a new historical stage of development. After the “12th 5-Year Plan,” China issued a number of laws on the construction of ecological civilization, and adopted “promoting the coordinated development of ecological civilization” as the content of the amendment to the national constitution, which has brought China's sustainable ecological and environmental protection undertakings into a stage of vigorous development. The State Council has made “guiding and managing ecological progress” as its function and power. Since then, China has established a basic institutional framework in terms of property rights of natural resource assets, development and protection of territorial space, total resource management, ecological compensation, improved environmental governance, and improved evaluation and accountability. A great progress has been made from national strategy to the building of a legal system, and from environmental supervision and evaluation to the participation of social forces.

**Table 5:** Decoupling of the efficiency of healthcare PPP projects and environmental pollution in China.

**Region**	**2011–2020**		**2011–2015**		**2015–2020**	
	**Decoupling elastic**	**State**	**Decoupling elastic**	**State**	**Decoupling elastic**	**State**
National	0.267	Weak decoupling	0.283	Weak decoupling	0.198	Weak decoupling
Northeast	1.08	Growth link	1.16	Growth link	0.99	Growth link
North China	0.399	Weak decoupling	0.426	Weak decoupling	0.311	Weak decoupling
Central China	0.052	Weak decoupling	0.136	Weak decoupling	0.0316	Weak decoupling
East China	0.177	Weak decoupling	0.234	Weak decoupling	0.147	Weak decoupling
South China	0.463	Weak decoupling	0.499	Weak decoupling	0.318	Weak decoupling
Southwest	0.872	Growth link	0.929	Growth link	0.787	Growth link
Northwest	1.33	Expansive negative	1.49	Expansive negative	1.25	Expansive negative
		decoupling		decoupling		decoupling

From the perspective of subregions, efficiency and environmental pollution in NEC show an increasing connection from 2011 to 2020. That is, the efficiency maintained a relatively synchronous growth trend with respect to environmental pollution.

In 2011–2020 and 2011–2015, SWC shows an increasing connection, indicating a synchronous growth trend but weak decoupling in 2015–2020, indicating less growth in environmental pollution with efficiency in the 13th 5-Year Plan.

From 2011 to 2020, NWC shows negative decoupling, that is, the growth rate of efficiency was less than the rate of increase in environmental pollution. In the same period, efficiency in NC, CC, EC, and SC show weak decoupling, that is, the growth rate of environmental pollution was less than that of efficiency.

The transformation of China's view of economic development and the requirement of socioeconomic development pose new challenges to PPP in healthcare and medicine. An ecology-oriented development (EOD) can possibly boost the quality and sustainability of medical PPP. Such a model is innovative in both the organization and implementation of projects. It prioritizes ecology and green development in regional and overall economic development. Better convergence is possible by absorbing this model to help achieve China's sustainability goals in PPP.

Environmental pollution is an inevitable problem for any country/region in the process of industrialization. However, the environmental dilemmas and governance difficulties encountered by different countries/regions are quite different due to the differences in economic and social structures. Therefore, in 2011–2020, the decoupling effect between the efficiency of medical PPP projects and environmental pollution is different in the different regions of China.

## Discussion

### Improvement in the Efficiency and Benefits of Medical PPP

According to the results of the analysis shown in the previous section of this study, it can be seen that some medical PPP projects are in a state of inefficiency; therefore, it is necessary to further improve the efficiency and benefits of medical PPP projects.

(1) Innovate and plan the early stages of medical PPP project

Planning is, in the early stage of a project, very important. The reason of many medical projects having problems in the implementation stage is due to problems in the early stage of planning. To implement a medical PPP project, its idea needs to be clarified, not just for the PPP model, but for the medical project using that PPP model, on the basis of doing a good job in the feasibility study and early planning of the project.

Firstly, medical projects should be selected, and reasonable arrangements should be made to build the time sequence. A PPP model should be adopted in accordance with the economic and social development needs, medical gaps, population health, and other aspects, a comprehensive consideration to priorities, and a reasonable project implementation timeline (57). Secondly, innovation should be used to improve the planning quality of medical projects. A single medical project can adopt a PPP mode, and a number of substantially related medical projects can also be included with it. In practice, new and stock projects can be packaged separately or together. However, it should be noted that the packaging must have a substantial correlation, and the scale should be moderate.

(2) Focusing on the construction of “Belt and Road” and learning from the experience of Guangdong, Hong Kong, and Macao cross-border PPP

Empirical results show that an uneven distribution of medical resources affects the coordinated development and efficiency of medical PPP between the regions. Two problems have plagued medical infrastructure investments for a long time: a large initial investment amount and long investment recovery period. Fundraising and investment are the important foundations for the implementation of medical infrastructure construction projects. Focusing on the “Belt and Road” construction, making the full use of foreign capital and foreign investment in medical institutions will help fill the vacancies of large private general hospitals in remote and backward areas in China, complement public hospitals, effectively improve medical service capabilities, and meet the diverse needs of the people. We can learn from the successful experience of the Guangdong-Hong Kong-Macao Greater Bay Area and that of “Belt and Road” countries, adhere to open cooperation, support multichannel funding to participate in infrastructure and long-term financing, and adhere to the openness of the “Belt and Road” cooperation.

With the accelerated reform of the global governance system and the international order, major breakthroughs have been made in comprehensively deepening reforms, and the construction of the “Belt and Road” is advancing rapidly, opening up new opportunities for the Guangdong-Hong Kong-Macao Greater Bay Area to participate in higher-level international cooperation and competition, and enhancing its competitiveness. In the past 6 years, as the “Belt and Road” initiative was put forward, the Guangdong-Hong Kong-Macao Greater Bay Area has created a good opportunity of jointly participating in and becoming an important support for the construction of “Belt and Road” projects.

The Guangdong-Hong Kong-Macao Greater Bay Area and the “Belt and Road” countries adopted a cross-border PPP model, which deeply binds international and domestic social capital and government interests (58). This enables the government and social capital to give play to their respective advantages, enhance the enthusiasm of both parties, and identify common goals. While resolving government debt risks and achieving VFM, they can achieve a win–win situation between the government and the market, making good use of the flexible and innovative policy mechanisms of the Greater Bay Area, revitalizing the land and other resources in the region, allocating a large amount of active social capital, introducing foreign capital, and driving the output of production capacity, with the goal of coordinated industrial development, urban prosperity, and fiscal revenue increase in Southeast Asian countries. To realize this model of sustainable self-sufficient development, the government, enterprises, and countries along the “Belt and Road” have established a community relationship of “benefit sharing, risk sharing, and full cooperation” in infrastructure such as pensions and other public services, medical and healthcare, with the goal of long-term cooperation in the field of construction and operation to achieve a mutual benefit and win–win results.

### Life Cycle Management of Medical PPP Projects

According to the analysis in the previous section of this study, when considering the environmental pollution of expected outputs, the efficiency of PPP projects changed. Thus, when evaluating the efficiency of medical PPP projects, the complexity, volatility, and input and output stages should be considered. A comprehensive life cycle performance management of medical PPP projects is an effective means to promote the standard, efficient, and high-quality operation of medical PPP projects (59). The VFM of medical PPP projects is an evaluation tool to demonstrate the best benefits of PPP model efficiencies (60), cost, and other aspects. Integrating the quantitative and qualitative evaluation of VFM of medical PPP projects into the performance management assessment of the whole life cycle of a medical PPP project is conducive to strengthening the supervision and management of the entire life cycle of the project, timely correction, and improvement. It empowers projects to achieve maximum benefits and efficiencies (61). The following points should be highlighted.

#### The Matching Degree of Scheme Design in the Early Stage Should Be Improved

The combination of VFM dynamic evaluation and performance management in the whole life cycle of medical PPP projects is a relatively new management concept. On one hand, it improves the VFM of medical projects, and on the other hand, it indirectly promotes the development of performance management. There are time differences in the evaluation indicators of the abovementioned tools. Therefore, to implement dynamic VFM evaluation in the full life cycle performance management of medical PPP projects, it is necessary to consider the matching degree of the two performance indicators in the pre-plan design of the project.

#### The VFM Indicator Should Be Included in the Performance Assessment of Medical PPP Projects

The performance objectives of medical PPP projects should conform to the concept of VFM and reflect cost-effectiveness. In order to consider the performance evaluation indicators of the whole life cycle of PPP projects and the degree of implementation, each stage of VFM should include a hard evaluation indicator system, focusing on the output, effect, management and other factors of the project. This would be conducive to a sustainable and an effective play of the VFM concept throughout the entire life cycle of medical projects.

### The Combination of PPP+EOD Mode in the Medical Field Should Be Explored

According to the results of our analysis, it can be seen that the efficiency of medical PPP in some regions of China is still increasing in a direct proportion to environmental pollution. The combination of the PPP+EOD mode should be explored in the medical field, to promote the low-carbon (62), green, high-quality development of medical PPP.

The transformation of economic development in China and the transformation of the economic and social development needs introduced new challenges to the development of medical PPP modes. To cope with these challenges, it is necessary to learn from the EOD model and concept, to boost the high-quality and sustainable development of China's medical PPP. The EOD mode is an innovative way of project organization and implementation, which adheres to ecological priorities, practices the concept of green development, and aims to promote high-level protection of ecological environment and high-quality development of the regional economy (63). This coincides with a high-quality sustainable development goal of PPP in China, and can be better modified on the basis of learning and absorbing the concept of the EOD mode to help achieve the goal of medical PPP development.

The combination of the EOD concept and the PPP mode is called PPP+EOD mode, which is similar to the development of the PPP mode. It takes into account the overall regional development as a platform and self-hematopoiesis and value-added benefits as the profit mechanism, and integrates the ecological concept into the whole life cycle of PPP projects, including every link and the entire process of planning and design, investment and construction, operation and management so that the ecological concept can produce practical benefits and achieve the goal of sustainable regional development. The PPP+EOD mode is new and should be further actively explored and implemented to promote the innovative development of China's medical PPP.

In future environmental governance work, the government should deeply integrate the concept of environmental protection into the construction of medical infrastructure and the layout of the medical industry, establish a multilevel and three-dimensional environmental governance system, and comprehensively supervise the pollution and resource consumption problems generated in the construction of medical infrastructure. Once the persistent pollution of medical projects occurs, the source of pollution can be accurately located and pollution responsibility can be implemented. An efficient implementation of environmental governance requires the adherence to a high-quality and well-coordinated development and improved overall coordination between urban environment and regional medical development. It attaches a great importance to the environmental management system in synergy with different subjects through an efficient integration of resources and the application of high-tech achievements, to realize the simultaneous improvement of environmental governance and regional medical standards. Similarly, integrating the prevention, monitoring, restoration and reconstruction of urban environmental governance systems and a clear division of labor and responsibilities should be established to accelerate the development of urban environmental governance efficiency and regional medical level high coordination.

### Public Participation in Medical PPP Projects to Jointly Tackle Environmental Pollution and Population Health Problems

The PPP theory aiming at sustainable development includes two aspects: “people first” and “public participation”, They are of great significance in dealing with environmental pollution and population health issues, establishing a reasonable PPP public participation mechanism, improving and effectively supervising project progress, and promoting the sustainability of medical PPP projects.

#### Improving Information Release Procedures to Protect the Right to Know

The government information disclosure in the medical PPP mode is improved: firstly, the focus should be on the monitoring and analysis of environmental indicators as it is necessary to actively disclose the demonstration information and to evaluate and monitor this information, in combination with the local environmental and ecological reality, before the project is launched; secondly, in the bidding stage of the project, the environmental pollution index, pollutant discharge capacity, the responsibility of pollution prevention and control all shall be taken as the entry threshold for bidding (64). Additionally, before the government and private enterprises reaching an agreement, they may consider establishing in advance a public review mechanism to encourage the public to participate in the proposal and vote on the bidding enterprise, with the voting result directly affecting whether the enterprise wins the bid or not.

#### Making It Mandatory for Companies to Disclose Environmental Information in the Project Operation Stage

Firstly, the two modes of voluntary and mandatory disclosure are combined. When it comes to trade secrets, the regulatory authority should make rulings based on whether they will infringe upon the environmental interests of the public. Secondly, the time for publication should be advanced to the project bidding stage. When companies participate in bidding, they should be required to disclose the same information to both the government and the public (65). Thirdly, the public should not only be able to require companies to directly disclose relevant environmental information, but they should be able to even require project sponsors to disclose relevant environmental information, to broaden the public's channels for information disclosure. A decision-making participation platform to broaden participation channels should be built.

#### Establishing a Forum for Environmental Decision-Makers

The most effective way to change the environmental problems caused by previous government-led project decisions is to build a third-party decision-making platform, which can directly influence the decision regarding the necessity of medical projects and the start of projects in the PPP mode, achieve scientific and democratic decision-making in health-related PPP projects, and prevent public's resentment especially as it relates to contradictory information. This would enable all PPP stakeholders to achieve a harmonious face-to-face, two-way communication, and change the way of organization of past government activities, in a mostly unidirectional manner, to create an advocacy-based model.

#### Leveraging the Internet to Build a Unified Information Collection Platform

Before making a decision on a medical PPP project, an internet application should be built to serve as an environmental information platform. Through this system, project information such as the government's review of the medical PPP environmental issues and project bidding company information could be released to local residents, ensuring their right to participate in the project decision-making. Similarly, local residents could input personal opinions directly into the system. Based on statistics, these opinions could be put forward at stakeholder meetings for reference. In case of objections to these opinions, the government representatives could provide an appropriate explanation.

#### Establishing a Feedback Mechanism for the Results of Environmental Public Participation

The realization of the right to participate not only requires personal participation in the decision-making process but also allowing the public to know the effects of their participation. After a decision is made, the outcomes and reasons for accepting or not accepting public opinions should be announced, focusing on aspects with relatively large differences, and public opinions should be solicited again. The forum for representatives could serve as an avenue for this process.

#### The Establishment of Multichannel Full Supervision to Achieve the Right to Request

Third-party non-governmental organizations (NGOs) should be given the right to centralize supervision. The public welfare and organizational nature of NGOs determine that they can gather the interests of the public and supervise environmental issues in the PPP model in an orderly manner. NGOs organizations should have the right to use their platforms to solicit public opinion and represent direct stakeholders to report environmental issues to the Environmental Protection Department. In response to the potential inaction of the Environmental Protection Department, appropriate legislation can be made to clarify the qualifications of the NGOs organizations and to initiate administrative reconsideration and administrative litigation (68). Finally, in response to the potential failure of government-sponsored departments to supervise medical PPP projects, the rights and procedures for NGOs organizations reported to higher levels should be clarified.

## Conclusion

To strengthen weak links and carbon emission double pressing requirements, there are still many challenges on the road to the development of medical PPP projects in China. How to achieve the continuous growth of medical PPP projects while ensuring their low-carbon footprint being an important topic, which is needed to be discussed urgently. However, the existing research has yet to consider environmental protection scenarios comprising medical PPP overall efficiency evaluations and changes to low efficiency projects, and to study the decoupling between them and environmental pollution.

To make up for this gap, we collected public historical data and used a three-stage DEA model to measure the efficiency of China's healthcare PPP projects from 2011 to 2020. The DEA efficiency measurement results in the first stage show that the efficiency of most medical PPP projects in scope is low and overall shows a decreasing trend, and that there are regional disharmony and imbalance in the geographical and temporal distribution of efficiency. Furthermore, using the SFA method to calculate the separating environment variable, the random error, and the influence of the internal management inefficiency results, our results show that air and water quality, environmental, and agricultural disaster factors will affect the output of medical PPP, namely regional population mortality rate and health status, subsequently affecting the efficiency of the PPP. In the third phase, after the adjustment of medical efficiency values and before the improvement of the PPP project, the visible environment factors have an effect on the efficiency of medical PPP. This enlightens us how to evaluate the efficiency of PPP projects: in addition to considering the expected output, considering also the efficiency of the evaluation results would be more scientific, reasonable, and comprehensive.

Based on the efficiency measurement results of the third-stage medical PPP, the decoupling effect between a variation of the medical PPP efficiency and environmental pollution was discussed. The results of the decoupling analysis show that, from 2011 to 2020, environmental pollution increases with an increase in the efficiency of China's healthcare PPP projects, but the growth rate of environmental pollution is smaller than that of the efficiency of the healthcare PPP projects. Compared with the variation range of the medical PPP efficiency, an increase in environmental pollution during the 13th 5-Year Plan period is smaller compared with the 12th 5-Year Plan period, which shows that efforts of China in environmental protection and economic transformation, upgrading, and development have achieved certain positive results. However, the decoupling effect between the efficiency of medical PPP and environmental pollution is not balanced among regions, indicating that the progress of carbon emission reduction and the efforts of environmental protection are asynchronous across the different regions of China. China's medical industry is carbon neutral, and the speed and intensity of carbon emission reduction need to be further strengthened.

Inevitably, there are some limitations to our study. Firstly, it failed to go deep into the provincial and municipal level. Secondly, the comprehensiveness and diversity verification of medical PPP input and output data still need to be improved. The comprehensiveness and diversity of medical PPP data need to be further improved. Future research can adopt multimodal machine learning methods to synthesize various databases to obtain more accurate and detailed evaluation results of medical PPP efficiency at provincial and municipal levels.

## Data Availability Statement

The original contributions presented in the study are included in the article/supplementary material, further inquiries can be directed to the corresponding author/s.

## Author Contributions

C-CC: conceptualization, methodology, and software. Z-HZ: data curation and writing—original draft preparation. BS and Y-YM: regression and writing—original draft preparation. Z-JW: writing—reviewing, data analysis, and visualization. All authors contributed to the article and approved the submitted version.

## Conflict of Interest

The authors declare that the research was conducted in the absence of any commercial or financial relationships that could be construed as a potential conflict of interest.

## Publisher's Note

All claims expressed in this article are solely those of the authors and do not necessarily represent those of their affiliated organizations, or those of the publisher, the editors and the reviewers. Any product that may be evaluated in this article, or claim that may be made by its manufacturer, is not guaranteed or endorsed by the publisher.
